# Challenges, Advances, and Future Directions in Nipah Virus Vaccine Development

**DOI:** 10.3390/vaccines14070584

**Published:** 2026-06-30

**Authors:** Hongshan Xu, Xuanxuan Zhang, Shuai Shang, Fangxuan Chen, Xinyu Liu, Qunying Mao

**Affiliations:** 1 National Institutes for Food and Drug Control, Beijing 102629, China; xuhongshan@nifdc.org.cn (H.X.); zhangxuanxuan@nifdc.org.cn (X.Z.); liuxinyu@nifdc.org.cn (X.L.); 2 Changchun Biological Products Research Institute Co., Ltd., Changchun 130012, China; shangshuai2026@163.com (S.S.); chenfangxuan1211@163.com (F.C.)

**Keywords:** Nipah virus, vaccine development, antigen design, technical platform, public health, Animal Rule

## Abstract

Nipah virus (NiV) is a highly pathogenic zoonotic pathogen. Since its discovery in 1998, recurrent epidemics have occurred in South and Southeast Asia, with a case fatality rate ranging from 40% to 100%. The outbreak in West Bengal, India in early 2026 has once again highlighted its severe threat to public health. To date, no licensed human vaccines or specific therapeutics against NiV are available worldwide. This review systematically summarizes the breakthroughs in antigen design for NiV vaccines, with a focus on conformational stabilization of prefusion F (pre-F) protein, chimeric G/F antigens, and multivalent nanoparticle strategies. In addition, we comparatively analyze the clinical progress of mainstream vaccine platforms, including viral vectors, mRNA and subunit vaccines. Given the sporadic nature and high mortality of NiV infection, the conventional licensing pathway relying on large-scale phase III clinical trials faces substantial practical obstacles. Accordingly, this article discusses adaptive adjustments in regulatory science. We propose several strategies to accelerate the clinical translation and emergency stockpiling of NiV vaccine candidates, including establishing unified correlates of protection thresholds, coordinating multinational regulatory resources, and optimizing the implementation of Animal Rule.

## 1. Introduction

Nipah virus (NiV) is a highly pathogenic zoonotic paramyxovirus belonging to the genus Henipavirus. It was first isolated in Malaysia in 1998, and recurrent outbreaks have since occurred across South and Southeast Asia, with reported case fatality rates ranging from 40% to 100% [1,2]. Classified as a Biosafety Level 4 (BSL-4) pathogen [3,4], NiV has been designated by the World Health Organization (WHO) as a high-priority emerging threat. Fruit bats are recognized as the natural reservoir of NiV. The virus can spill over to humans either via intermediate hosts such as pigs and horses or through direct contact, frequently causing severe encephalitis and acute respiratory distress. Sustained human-to-human transmission poses a potential risk of global pandemic [1,2].

Although the annual incidence of NiV infection worldwide remains generally low, with fewer than 100 confirmed cases reported each year [5], the epidemiological characteristics of NiV have evolved over time. South Asia has now become an endemic region for NiV, where outbreaks are recorded almost annually in Bangladesh and India [6]. In January 2026, a cluster of NiV cases emerged in West Bengal, India, involving multiple healthcare workers and highlighting the substantial risk of nosocomial transmission [7,8,9,10]. As of June 2026, a total of 765 confirmed cases and 441 deaths have been documented in the Asia–Pacific region [11,12,13,14,15,16,17] (Summarize statistical data based on references [11,12,13,14,15,16,17]). The rising frequency of outbreaks underscores the urgent need to develop safe and effective vaccines against NiV.

Vaccination is widely acknowledged as one of the most cost-effective interventions for the prevention of viral diseases. Since the early 2000s, multiple NiV vaccine candidates, including ChAdOx1-NiVb, PHV02, mRNA-1215 and HeV-sG-V, have advanced into clinical development [18,19,20,21,22,23,24]. Nevertheless, none of these candidates have obtained regulatory approval to date. The clinical translation of NiV vaccines is hindered by multiple challenges, including sporadic outbreaks [2,5,6], inadequate correlates of protection (CoP) [25,26], mandatory BSL-4 containment facilities, discrepancies in pathogenic mechanisms between animal models and humans [27,28,29], inconsistent global regulatory frameworks [27,30], and limited commercial incentives [31,32,33].

This review systematically summarizes recent advances in the pathogen biology and epidemiology of NiV, as well as the antigen screening strategies for NiV vaccine development. We also compare the latest clinical progress of major vaccine platforms, such as viral vectors, mRNA and recombinant subunits, and comprehensively analyze the multifaceted challenges facing NiV vaccine research. This work aims to present an up-to-date and holistic overview of NiV vaccine development, provide references for future research directions, and strengthen global public health preparedness and emergency response capacity against NiV outbreaks.

## 2. Virology

NiV is an enveloped, helical, non-segmented negative-sense single-stranded RNA virus with a genome of approximately 18.2 kb. Its genome sequentially encodes six structural proteins in the 3′-to-5′ orientation, including nucleocapsid (N), phosphoprotein (P), matrix (M), fusion glycoprotein (F), attachment glycoprotein (G), and large RNA-dependent RNA polymerase (L) [34]. The P gene further encodes three accessory proteins (V, W, and C) through RNA editing and alternative translational initiation. As pivotal virulence determinants of NiV, these accessory proteins antagonize host innate immune responses via the suppression of interferon (IFN) signaling, thereby facilitating viral immune evasion [35,36]. The N protein encapsidates viral genomic RNA to form ribonucleoprotein (RNP) complexes, which cooperate with the L-P polymerase complex to constitute the functional template for viral transcription and replication. The M protein anchors viral RNP complexes to envelope-embedded F and G glycoproteins and is therefore essential for the assembly and budding of mature infectious virions [37].

NiV belongs to a single serotype and comprises two major genetic lineages: the Malaysian lineage (NiV-MY) and the Bangladeshi lineage (NiV-BD) [4]. The surface G and F glycoproteins are highly conserved between the two lineages, with amino acid identities of approximately 96% and 95%, respectively [38]. NiV-MY and NiV-BD share high genomic similarity, with most mutations mapping to non-essential antigenic regions. This genomic feature supports the development of universal NiV vaccines capable of conferring broad cross-lineage protection. Nevertheless, subtle lineage-specific sequence variations substantially alter the binding affinity of monoclonal antibodies, highlighting the necessity of refined antigen design, standardized immunogenicity evaluation, and unified reference criteria for NiV vaccine development [38,39].

### 2.1. Surface Glycoproteins: Primary Targets of Neutralizing Antibodies

NiV envelope G and F glycoproteins represent the primary targets of host neutralizing antibodies and are the most translationally viable antigens for the development of novel henipavirus vaccines. As a homotetrameric attachment protein, G mediates viral adsorption and entry through specific binding to host ephrin-B2 and ephrin-B3 receptors. The G protein extracellular domain consists of a membrane-proximal stalk and a C-terminal globular head that adopts a canonical six-bladed β-propeller fold, which harbors major epitopes recognized by broadly neutralizing antibodies [37,40]. Structural and functional investigations have validated the G protein head domain and pre-F extracellular domain as the core scaffolds for rational NiV vaccine design. Multivalent antigen display strategies markedly improve vaccine immunogenicity and expand cross-protective coverage against heterogeneous NiV strains, demonstrating considerable potential for innovative vaccine development [8]. Spontaneous mutations in the native G protein are rare, whereas site-directed mutagenesis has advanced the mechanistic understanding of receptor engagement, F-mediated membrane fusion activation, and viral immune evasion [38].

The F glycoprotein assembles into envelope-resident homotrimers and mediates fusion between viral and host cellular membranes, a prerequisite for viral genome delivery and productive infection. F protein is initially translated as the inactive F0 precursor, which undergoes proteolytic processing by host endosomal cathepsins to generate two disulfide-linked functional subunits (F1 and F2) with full membrane fusion competence [41]. The prefusion conformation of the NiV F trimer is metastable and displays a dendrite-like architecture composed of three discrete structural domains (D1–D3). Receptor engagement triggers irreversible conformational rearrangement of the F protein, culminating in the formation of a thermostable post-fusion six-helix bundle [8,39]. Such structural remodeling generates mechanical force that drives membrane merging and facilitates viral entry into target cells.

F protein fusogenicity is precisely modulated by dynamic intermolecular crosstalk between G and F proteins. Receptor binding induces progressive conformational transitions in the G protein, propagating from the globular head to the membrane-proximal stalk and triggering structural remodeling of adjacent F trimers. This process releases the fusion peptide, which inserts into the host membrane and initiates a cascade of fusion events. Concurrently, two conserved heptad repeat motifs (HRA and HRB) within the F extracellular domain undergo spatial rearrangement to form a compact six-helix bundle, which approximates viral and cellular membranes and ultimately completes membrane fusion [15,39,40].

Structural stabilization of the F protein prefusion conformation constitutes a pivotal advance in NiV vaccine research. Site-specific disulfide bond introduction and proline substitutions enable conformational locking of F protein in its native prefusion state, thereby preserving conformation-dependent neutralizing epitopes that are lost during post-fusion structural rearrangement [39]. Systematic mutational analysis has identified 14 critical residues within the F extracellular domain responsible for prefusion stabilization, which are categorized as either structural determinants of fusogenicity or immunodominant hotspots targeted by cross-lineage neutralizing antibodies [42]. A total of nine monoclonal antibodies targeting pre-F protein have been isolated from rhesus macaques, four of which exhibit potent cross-neutralizing activity against multiple henipaviruses. Combined application of anti-F and anti-G monoclonal antibodies yields synergistic neutralizing effects. Structural characterization indicates that these protective antibodies adopt an IGHV4-59 germline framework with an elongated heavy chain complementarity-determining region 3, which occupies inter-subunit cavities within the F trimer and obstructs irreversible pre-to-post fusion transition, thereby stabilizing the immunodominant prefusion conformation [42]. These structural findings provide a mechanistic basis for the rational design of NiV vaccines and fusion-targeted therapeutic antibodies.

### 2.2. Structure and Function of the Polymerase Complex

Viral transcription and replication are catalyzed by the NiV L-P polymerase complex, consisting of the catalytic L protein and its essential cofactor P protein. The L protein harbors canonical RNA-dependent RNA polymerase activity, while the P protein functions as a processivity factor to sustain efficient and continuous viral RNA synthesis [8]. Recent cryo-electron microscopy studies have resolved the conserved architecture of the NiV L-P complex, which exhibits high structural homology with polymerases of other non-segmented negative-sense RNA viruses [43,44]. Although the L-P complex lacks prominent neutralizing epitopes and is not a direct vaccine target, its resolved structural features facilitate the development of recombinant viral vectors and minigenome platforms, supporting preclinical functional evaluation of NiV vaccine candidates [44].

### 2.3. Roles of Matrix Protein and Viral Inclusions

The M protein serves as a central regulator of NiV morphogenesis, assembly, and budding. It bridges intracellular RNP complexes with membrane-anchored F and G glycoproteins, thereby coordinating virion assembly and egress [37,45]. NiV infection induces the formation of two functionally distinct cytoplasmic inclusion bodies. Cytoplasmic inclusions serve as the primary site for viral RNA synthesis, whereas membrane-proximal inclusions are specialized for progeny virion assembly. The formation and functional segregation of these inclusions rely on synergistic interactions among N, P, and M proteins [37]. Despite lacking dominant neutralizing epitopes, the M protein-mediated assembly mechanism provides fundamental theoretical support for the design of NiV virus-like particle (VLP) vaccines. Eukaryotic co-expression of M, F, and G proteins enables spontaneous self-assembly of NiV VLPs. These non-infectious particles structurally mimic native virions and retain robust immunogenicity, representing promising NiV vaccine candidates with substantial translational potential [45].

### 2.4. Non-Structural Proteins and Viral Immune Evasion

NiV employs sophisticated immune evasion mechanisms to antagonize host innate antiviral defenses, which are primarily governed by three P gene-encoded accessory proteins (V, W, and C). V and W proteins are generated via P gene RNA editing and function as potent IFN antagonists. Both proteins possess an identical N-terminal STAT1-binding domain, whereas divergent C-terminal sequences confer distinct subcellular localization and immune regulatory functions. Cytoplasm-resident V proteins sequester STAT1/STAT2 complexes and suppress their tyrosine phosphorylation, thereby inhibiting JAK-STAT signaling transduction and downstream IFN responses [46]. In contrast, nucleus-localized W proteins promote cytoplasmic STAT1 nuclear translocation and repress the transcription of interferon-stimulated genes (ISGs) [46]. The C protein further potentiates viral immune evasion through the suppression of host IFN production [35].

Accumulating evidence has demonstrated that the NiV-BD W protein contains a unique C-terminal nuclear localization signal that sustains stable nuclear accumulation. Nuclear W protein inhibits the phosphorylation and accumulation of interferon regulatory factor 3 (IRF3), specifically ablates Toll-like receptor 3-triggered antiviral signaling, and ultimately represses IFN-β promoter activation. This immunosuppressive phenotype is exclusive to the nuclear W protein and is not observed for the cytoplasmic V protein. In addition, W protein interacts with karyopherin-α3 and 14-3-3 proteins to attenuate nuclear factor-κB-dependent proinflammatory responses, which constitutes a key molecular mechanism underlying the severe clinical outcomes of NiV infection [15,47]. The potent immunosuppressive capacity of NiV-BD enables efficient viral replication within respiratory epithelial cells, accelerates systemic viral dissemination, exacerbates disease severity, and increases the risk of sustained human-to-human transmission [4].

Strain-specific pathogenic traits provide critical guidance for the rational design of optimized NiV vaccines. Given that NiV-BD is the globally predominant epidemic strain characterized by high virulence and robust human transmissibility, vaccine antigen design should prioritize NiV-BD-derived sequences to maximize protective efficacy against circulating virulent strains. Furthermore, vaccine development strategies must account for the unique immunomodulatory properties of NiV-BD non-structural proteins, which alter viral replication kinetics, impair host innate immune activation, and shorten the durability of vaccine-mediated protection [29]. Collectively, the development of broad-spectrum, high-efficacy NiV vaccines requires targeted and refined design strategies. Optimal vaccine candidates should elicit potent neutralizing responses against NiV-BD surface glycoproteins while incorporating rational design principles to counteract viral immune evasion pathways, thereby improving public health preparedness against NiV outbreaks.

### 2.5. Rational Immunogen Design Strategies to Overcome Viral Immune Evasion

Rapid elicitation of high-titer neutralizing antibodies prior to the full activation of NiV immune evasion machinery is essential for blocking early viral invasion and establishing effective immune protection. Structurally stabilized pre-F proteins retain conformation-specific neutralizing epitopes that are irreversibly lost during post-fusion remodeling. Antibodies targeting these prefusion-restricted epitopes block viral entry at early infection stages, reduce the abundance of infected cells, and mitigate V/W-mediated IFN antagonism at the source. Preclinical studies have verified that pre-F-based immunogens induce 10–100-fold higher neutralizing titers than post-fusion or unmodified F proteins, with seroconversion achieved within 7–10 days post-immunization [22,23]. Rapidly induced robust humoral immunity enables neutralization of invading virions before the establishment of productive infection, substantially reducing host dependence on intact IFN signaling for antiviral defense.

Chimeric pre-F/G antigens (e.g., mRNA-1215) represent an advanced immunogen design that simultaneously displays multiple entry-blocking epitopes derived from both F and G glycoproteins. This design broadens the neutralizing antibody repertoire and cross-protective spectrum while reducing the propensity for viral immune evasion via antigenic drift. Since both F and G proteins are indispensable for viral entry, antibody targeting of either glycoprotein efficiently inhibits membrane fusion. Moreover, such chimeric immunogens induce high levels of functionally active antibodies with Fc effector potential, which mediate antibody-dependent cellular phagocytosis and complement deposition to eliminate infected cells even under IFN signaling-compromised conditions [22,23].

While humoral immunity primarily eliminates extracellular virions, cellular immunity is responsible for clearing NiV-infected cells and terminating persistent viral replication. Although NiV V and W proteins efficiently suppress STAT1/2-dependent IFN signaling, they do not directly disrupt major histocompatibility complex class I/II-restricted antigen presentation. Optimized composite immunogens (chimeric proteins or co-delivered mRNAs) based on F and G antigens elicit robust responses of CD4^+^ helper T cells, follicular helper T cells, and CD8^+^ cytotoxic T lymphocytes (CTLs) [22]. CTL cytotoxicity is mediated by perforin–granzyme exocytosis rather than STAT-dependent transcriptional regulation, rendering CTL effector function resistant to IFN pathway suppression. Even in the presence of complete type I/II IFN response abrogation by V/W proteins, CTLs retain the capacity to eliminate infected cells and halt viral propagation.

Follicular helper T cell activation further promotes germinal center formation and long-lived plasma cell differentiation, sustaining persistent high-affinity antibody production and durable immune protection. NiV V and W proteins suppress ISG expression and constrain the initiation of host adaptive immunity. Nevertheless, chimeric and multivalent immunogens bypass innate immune restrictions and provide sufficient co-stimulation for T cell activation, enabling the induction of long-term immune memory even in immunosuppressive microenvironments with impaired IFN signaling [22].

The synergistic interplay between high-titer neutralizing antibodies and robust CTL responses facilitates rapid viral clearance and alleviates V/W-mediated systemic immunosuppression. Viral kinetic modeling demonstrates that a 2–3 log reduction in peak viral load via vaccination substantially shortens the time window for viral accumulation of sufficient V/W proteins to paralyze host IFN defenses, thereby temporally restricting the execution of viral immune evasion [27]. Preclinical lethal challenge studies confirm that animals immunized with pre-F/G mRNA vaccines or chimeric adenovirus-vectored vaccines exhibit undetectable viral loads in all tissues, indicating that sterilizing immunity is established prior to the full activation of viral immune evasion programs [22].

In summary, structurally optimized NiV immunogens do not directly restore V/W-suppressed STAT1/2 or MDA5 signaling. Instead, viral immune evasion is overcome via accelerated adaptive immune activation, enhanced response magnitude, and diversified antigen recognition. This immunization strategy ensures that neutralizing antibodies and effector T cells achieve protective thresholds before the establishment of virus-induced systemic immunosuppression, thereby counteracting the pathogenic consequences of NiV immune evasion and providing a robust theoretical and technical basis for rational NiV vaccine development.

## 3. Epidemiology: Transmission, Diffusion and Evolutionary Dynamics of Two Lineages of Nipah Virus

### 3.1. Strain Lineage Differentiation and Phenotypic Variation

NiV is a highly virulent zoonotic paramyxovirus, with fruit bats of the genus Pteropus recognized as its primary natural reservoir. Long-term endemic circulation of NiV in bat populations is primarily driven by host adaptive evolution, which shapes the extensive genetic heterogeneity of field isolates. Phylogenetic reconstruction based on 66 full-length genomic sequences robustly classifies global NiV strains into two genetically distinct monophyletic lineages, namely the Malaysian lineage (NiV-MY) and the Bangladeshi lineage (NiV-BD) [4]. Pairwise genomic alignment reveals approximately 91.8% nucleotide identity between the two lineages, and molecular clock calibration dates their most recent common ancestor to approximately 1863 [48].

Long-term adaptive diversification within bat hosts has facilitated independent cross-species spillover of the two NiV lineages, leading to the inaugural human outbreaks in Malaysia (1998–1999) and Bangladesh (2001). Over two decades of post-divergence evolution, NiV-MY and NiV-BD have evolved stably maintained, phenotypically distinct epidemiological profiles that sustain endemic circulation across Southeast and South Asia.

Accumulated empirical evidence confirms pronounced lineage-specific disparities in pathogenicity, clinical manifestations and transmissibility between NiV-MY and NiV-BD. Aerosol challenge experiments in African green monkeys demonstrate distinct lethal outcomes, with a mortality rate of 27% for NiV-MY infection and 75% for NiV-BD infection. NiV-BD infection is further characterized by systemic viral dissemination and acute respiratory failure in infected animals [49]. Consistent with phenotypic variations, histopathological lesions differ substantially between lineages: NiV-MY predominantly induces systemic vasculitis and severe neurological encephalitis, whereas NiV-BD causes diffuse pulmonary hemorrhage and fibrin thrombosis, resulting in dominant respiratory tissue damage [49].

These lineage-specific pathogenic discrepancies are recapitulated in a Syrian hamster infection model. NiV-BD-infected hamsters develop prominent pulmonary lesions without apparent neurological dysfunction, accompanied by significantly elevated pulmonary interleukin-6 (IL-6) expression compared with NiV-MY-infected hamsters. These findings corroborate divergent immune activation patterns and tissue tropism underlying the pathogenic differentiation of the two NiV lineages [50].

Unlike the genetically stable NiV-MY lineage, NiV-BD undergoes persistent genetic diversification and sublineage differentiation in endemic regions of Bangladesh. Bayesian phylogeographic inference based on 23 nucleoprotein gene sequences derived from human and bat specimens collected during 2016–2023 indicates that contemporary Bangladeshi NiV-BD strains diverged into two basal monophyletic clades (BD1 and BD2) in 1984 [51]. Subsequent subdivision after 2008 generated four secondary sublineages, including BD1.1/BD1.2 derived from BD1 and BD2.1/BD2.2 derived from BD2. As of 2023, the BD2 sublineage has been identified as the predominant genotype circulating in local Bangladeshi bat populations.

Evolutionary rate quantification estimates a mean nucleotide substitution rate of 4.89 × 10^−4^ substitutions/site/year (95% highest posterior density [HPD]: 3.71 × 10^−4^–6.45 × 10^−4^) for NiV-BD. Spatial genetic analysis further reveals a geographical dispersal range of 6.54 km to 116.09 km, demonstrating that NiV-BD undergoes independent adaptive evolution in discrete geographical niches, continuously generating novel genetic variants and expanding viral population diversity [51].

The profound genetic and phenotypic heterogeneity between NiV-MY and NiV-BD poses substantial challenges for the development of pan-lineage NiV vaccines. Next-generation vaccine candidates must elicit robust, broad-spectrum cross-protective immunity against both prototype lineages. Considering the ongoing genetic drift and sublineage proliferation of NiV-BD, vaccine antigens should be rationally designed to target functionally indispensable and evolutionarily invariant viral domains to circumvent mutation-induced immune evasion.

Conventional NiV vaccine strategies primarily target surface glycoproteins, which are prone to antigenic variation and confer limited cross-protective efficacy. Recent advances in computational vaccinology have overcome this bottleneck by validating multiple multi-epitope vaccine candidates targeting the highly conserved viral RNA-dependent RNA polymerase (L protein). The inherent sequence conservation and low antigenic drift risk render the L protein an ideal pan-NiV vaccine target, broadening the conventional research paradigm limited to surface immunogens [9].

### 3.2. Transmission Routes and High-Risk Populations

NiV-MY and NiV-BD exhibit fundamentally divergent spillover ecology, host transmission networks and epidemic dynamics, supporting two completely independent zoonotic transmission cycles in Asia. The transmission cycle of NiV-MY is strictly dependent on domestic pigs as intermediate amplifying hosts, with no capacity for direct bat-to-human spillover or sustained human-to-human transmission. The 1998–1999 Malaysian NiV outbreak was initiated by cross-species spillover from fruit bats to domestic pigs, followed by secondary zoonotic transmission to farm and slaughterhouse workers via direct exposure to infectious porcine respiratory secretions and bodily fluids. The epidemic caused 265 confirmed cases and 105 deaths in Malaysia, with an additional 11 cases and one death in Singapore, yielding an overall case fatality rate (CFR) of approximately 40% [52,53]. The absence of secondary human transmission chains validates the inability of NiV-MY to sustain interpersonal dissemination, and large-scale pig culling operations successfully interrupted the transmission cycle and terminated the regional epidemic [52,53].

In stark contrast, endemic NiV-BD circulation in South Asia does not require livestock amplification intermediates. Primary human infections are predominantly attributed to direct exposure to raw date palm juice and wild fruits contaminated by bat excreta, while domestic pigs are not involved in the NiV-BD transmission cascade [54]. As of September 2025, a cumulative total of 347 NiV-BD cases have been officially reported in Bangladesh, with an overall CFR of 71.7%, which is markedly higher than that of NiV-MY-associated epidemics [55].

Epidemiological source tracing demonstrates that nearly 50% of primary NiV-BD infections in Bangladesh stem from the consumption of contaminated raw date palm juice, and approximately 33% of confirmed cases arise from secondary human-to-human transmission [54,56]. NiV-BD epidemics exhibit robust seasonal periodicity, with peak incidence occurring annually from December to April, which coincides precisely with the seasonal harvesting and consumption of date palm juice. This stable temporal pattern facilitates a self-sustaining zoonotic spillover and human transmission loop in Bangladesh [56].

Differential interpersonal transmissibility represents the most definitive phenotypic distinction between the two NiV lineages. Controlled ferret infection experiments provide direct in vivo evidence that NiV-BD-infected ferrets produce substantially higher viral loads in oral secretions than NiV-MY-infected animals. The enhanced oral viral shedding capacity underpins the efficient human-to-human transmission potential of NiV-BD, whereas NiV-MY lacks such transmissibility traits [57].

Human-to-human transmission of NiV-BD is predominantly clustered in household care and nosocomial settings, mediated by infectious respiratory droplets, saliva and urine, which frequently drive secondary epidemic amplification [56,58,59]. A nosocomial cluster outbreak in Barasat, West Bengal, India, in January 2026 caused confirmed infections in five healthcare workers (HCWs), highlighting substantial public health risks associated with cross-border dissemination and hospital-acquired NiV-BD transmission [60]. Strategic vaccine stockpiling, emergency ring vaccination, and targeted immunization of frontline HCWs and rural high-risk populations are recognized as the most viable interventions to contain NiV-BD outbreaks.

#### 3.2.1. Dual Epidemic Patterns of Nipah Virus in India

India is the only country worldwide that sustains dual epidemiological patterns of NiV-BD circulation. The West Bengal region, adjacent to the Bangladesh border, has experienced recurring cross-border NiV outbreaks, including the 2001 Siliguri outbreak (CFR = 68%) and the 2007 Nadia outbreak (CFR = 100%). Re-emergent local cases in 2026 further confirm the periodic outbreak pattern in this region over the past two decades [40,61].

The southern Indian state of Kerala has experienced sporadic NiV outbreaks since the index local case was identified in Kozhikode in 2018, with continuous case documentation from 2019 to 2025. Kerala’s epidemics are characterized by small-scale, localized clusters amenable to rapid containment without large-scale nosocomial spread. Despite the widespread geographical distribution of Pteropus bats across India, human NiV cases are strictly confined to West Bengal and Kerala. This heterogeneous distribution indicates that NiV cross-species spillover is not solely determined by reservoir distribution but is co-modulated by ecological exposure conditions, human population density, healthcare emergency response capacity and human–wildlife interface interactions [60,61].

#### 3.2.2. Subclinical Infection and Quantitative Analysis of Transmission Dynamics

NiV infection exhibits a broad clinical spectrum, and prevalent subclinical and asymptomatic infections significantly impede epidemiological tracing and epidemic prevention. During the 2018 Kerala NiV outbreak, serological screening was conducted on 279 close contacts of 18 laboratory-confirmed cases. Three subclinical infections were identified, including two individuals with dual IgM/IgG seropositivity and one individual with isolated IgM positivity, corresponding to an overall seroprevalence of 1.08% (95% confidence interval [CI]: 0.37–3.11%). All seropositive individuals remained completely asymptomatic throughout the exposure period [62].

A prospective household cohort study in Bangladesh revealed a high prevalence of subclinical NiV-BD infection. Among 110 household contacts of confirmed cases, 30 individuals (27.3%) developed symptomatic infection and six individuals (5.4%) presented subclinical seroconversion, yielding an aggregate household infection rate of 33% [63]. These findings indicate that conventional passive case surveillance markedly underestimates the actual transmission activity of NiV-BD in high-intensity close-contact scenarios.

Systematic reviews and meta-analyses have quantitatively synthesized core epidemiological parameters of NiV transmission, providing fundamental datasets for epidemic modeling and risk prediction [64,65]. Population serological surveys demonstrate that NiV IgG seroprevalence in endemic regions ranges from 0% to 12.5% with prominent geographical heterogeneity. The median incubation period of NiV infection is estimated at 8.77 days (95% CI: 7.53–10.02 days, *n* = 165).

Significant CFR variability exists across geographical regions and viral lineages. The NiV-MY outbreak in Singapore exhibited the lowest CFR at 9.1% (95% CI: 0.2–41.3%), while NiV-BD outbreaks in Bangladesh presented the highest CFR at 81.9% (95% CI: 71.9–88.9%) [64]. Most basic reproduction number (R_0_) estimates for NiV-BD are below 1, consistent with field observations that NiV-BD transmission is limited to short, discrete chains that typically terminate spontaneously after 2–4 transmission generations [64].

Transmission network analysis of 248 NiV cases recorded in Bangladesh during 2001–2014 quantified the heterogeneous transmissibility of NiV-BD, with each primary case generating an average of 0.23 secondary infections among close contacts (95% CI: 0.11–0.46) [66]. Secondary interpersonal transmission of NiV-BD is highly context-dependent and restricted to high-exposure household and nosocomial environments. Mild and asymptomatic community infections cannot sustain persistent viral dissemination, which explains why NiV-BD rarely causes nationwide large-scale epidemics despite its inherent human transmissibility [66].

#### 3.2.3. Quantitative Analysis of Transmission Routes and High-Risk Populations

Four predominant NiV transmission routes have been quantitatively validated based on multi-regional outbreak surveillance data accumulated over two decades, providing evidence-based support for precise epidemic intervention strategies.

##### Food-Borne Zoonotic Spillover (Bat → Fruit/Juice → Human)

This pathway represents the dominant zoonotic spillover route of NiV-BD. Statistical analysis of 2001–2023 epidemiological data shows that 45.72% (155/339, 95% CI: 40.33–51.19%) of NiV cases in Bangladesh are attributable to the consumption of bat-contaminated raw date palm juice and environmental exposure to bat-contaminated substrates [67].

Logistic regression risk modeling indicates that residents in villages with seasonal date palm juice harvesting during epidemic periods exhibit a 26.97-fold higher infection risk (95% CI: 5.98–121.67) than populations in low-exposure remote villages [68]. The annual harvesting season (December–April) overlaps with the peak foraging activity of local Pteropus bat populations. Furthermore, NiV remains viable in high-sugar date palm juice for several days, causing persistent environmental contamination and sustained zoonotic spillover risks [4,69].

##### Intermediate Host-Mediated Spillover (Bat → Livestock → Human)

Livestock-mediated transmission is exclusive to NiV-MY epidemics, with domestic pigs serving as the sole biological amplification host. The 1998–1999 Malaysian epidemic resulted in 265 pig-origin human infections with a CFR of 40% [59].

A distinct dose–response correlation exists between pig exposure intensity and infection risk. Personnel engaged in high-intensity swine breeding operations demonstrate a symptomatic infection rate of 51%, whereas low-exposure workers show an infection rate of 21%, representing a 2.4-fold risk discrepancy [70]. Large-scale culling of approximately 1.16 million pigs effectively disrupted the transmission cycle and terminated the regional epidemic [71]. Although sporadic livestock spillover events have been documented in NiV-BD-endemic regions, domestic pigs are not involved in the natural transmission cycle of NiV-BD [70].

##### Direct Non-Food-Borne Bat-to-Human Transmission

Direct contact with bat secretions and excreta constitutes a supplementary sporadic spillover pathway. Epidemiological surveillance in Bangladesh confirms that 33.33% of outbreak sites harbor resident Pteropus colonies within a 10-km radius, with 18.80% of environmental samples from bat habitats testing positive for NiV nucleic acid [67].

Virological detection reveals a 0.45% NiV positivity rate in bat saliva samples (1/224), and serological surveillance shows a bat population seroprevalence of 15.2% (95% CI: 10.74–20.56%) at local roosting sites [67]. These data indicate that despite widespread viral carriage in reservoir bat populations, cross-species spillover is a low-probability event requiring synergistic interactions between specific ecological conditions and human high-risk behaviors.

##### Human-to-Human Clustered Transmission (Nosocomial and Household Transmission)

Secondary human-to-human transmission is the primary driver of NiV-BD epidemic amplification and the key phenotypic distinction from NiV-MY. From 2001 to 2023, 66.37% (225/339, 95% CI: 61.08–71.40%) of confirmed cases in Bangladesh were clustered secondary infections originating from primary zoonotic spillover events [67]. NiV-BD interpersonal transmission exhibits prominent clustering and superspreading heterogeneity. Epidemiological tracing shows that 51% of secondary cases occur in family members and non-professional caregivers, and 9% in frontline HCWs [72,73]. Mathematical modeling validates that 92.0% (95% CI: 87.0–95.4%) of primary cases do not generate secondary infections, and epidemic expansion is predominantly driven by rare superspreading events [74]. Advancements in seroepidemiological techniques enable precise risk stratification of NiV-exposed populations, optimizing targeted prevention and control strategies. The risk characteristics of major high-risk groups are summarized as follows.

Healthcare workers: Meta-analysis of 16 multinational cohort studies shows that the pooled NiV IgG seroprevalence among endemic HCWs is as low as 0.08%, whereas the pooled CFR following infection reaches 73.52% (95% CI: 34.01–99.74%) [75]. Substandard personal protective practices in resource-limited medical settings are the primary contributor to high mortality. The 2026 nosocomial outbreak in India further corroborates the severe occupational exposure risk faced by frontline HCWs [75].

Swine industry workers (NiV-MY-endemic areas): Infection risk is positively correlated with swine contact intensity. Workers engaged in high-load swine breeding operations exhibit a 2.4-fold higher infection risk than low-exposure personnel, highlighting the pig–human interface as a core surveillance priority for NiV-MY prevention [70].

Date palm juice harvesters and consumers (NiV-BD-endemic areas): A large proportion of local residents in endemic Bangladesh regularly engage in seasonal date palm juice harvesting and consumption, with frequent exposure to bat-contaminated wild fruits [67]. Consumption of raw date palm juice (adjusted odds ratio [aOR] = 3.96, 95% CI: 3.5–4.4) and residence within 5 km of bat roosting sites (aOR = 1.18, 95% CI: 1.02–1.37) are two independent risk factors for NiV zoonotic spillover [67,68].

Rural and suburban residents in endemic regions: Population serological surveys across South and Southeast Asia reveal NiV IgG seroprevalence ranging from 0% to 12.5%, with the highest seropositivity detected in seasonally endemic areas of Bangladesh [8,76]. High-risk spillover zones are characterized by overlapping geographical distributions of bat habitats, date palm plantations, human settlements and livestock breeding sites, forming high-risk ecological interfaces for zoonotic viral transmission [77,78].

The transmission routes, epidemic patterns and high-risk populations of NiV are systematically summarized in Table 1.

### 3.3. Implications for Vaccine Development and Immunization Strategies

Cumulative findings regarding NiV viral evolution, phenotypic pathogenesis and transmission dynamics provide robust theoretical and epidemiological evidence for rational vaccine design and optimized immunization deployment strategies.

First, the extensive genetic and phenotypic divergence between NiV-MY and continuously diversified NiV-BD sublineages mandates the development of next-generation vaccines with potent, broad-spectrum cross-protective efficacy against all circulating NiV genetic variants.

Second, the ongoing adaptive evolution and sublineage proliferation of NiV-BD expose the inherent limitations of conventional vaccines targeting variable surface glycoproteins, which are susceptible to antigenic drift and subsequent immune escape. The evolutionarily conserved viral L protein has emerged as an optimal alternative target for multi-epitope vaccine design, which effectively avoids mutation-mediated immune evasion and expands the existing framework of NiV vaccine research [67].

Third, quantitative transmission dynamic parameters enable evidence-based, precise vaccine allocation. Given the persistent secondary interpersonal transmission and recurrent sporadic outbreaks in endemic regions, strategic national vaccine stockpiling, emergency ring vaccination and targeted immunization of high-risk populations represent the most cost-effective and operationally feasible intervention measures.

Furthermore, the unique epidemiological features of NiV, including high fatality, low incidence and sporadic clustered outbreaks, preclude the implementation of conventional large-scale Phase III clinical efficacy trials. Therefore, the vaccine evaluation paradigm should be shifted from traditional clinical endpoint verification to immunological biomarker-based efficacy inference. Broad-spectrum protective efficacy against heterogeneous NiV-BD strains and rapid immune onset should be prioritized as core preclinical evaluation indices. In practical public health applications, targeted pre-emptive immunization for high-risk groups and emergency ring vaccination strategies are more pragmatic and efficient than universal population-wide immunization.

## 4. Research Status and Progress of Nipah Virus Vaccines

Global scientific institutions have continuously advanced the research and translational application of prophylactic vaccines against Nipah virus, relying on four core technical systems: viral vector, messenger ribonucleic acid (mRNA) and recombinant subunit vaccine platforms. As of early 2026, four candidate vaccines developed through the aforementioned technical routes, including chimpanzee adenovirus-vectored, vesicular stomatitis virus (VSV)-vectored, mRNA, and recombinant subunit vaccines, have progressed to clinical trials. Among these investigational products, ChAdOx1 NipahB, which is designed to target genotype B Nipah virus, represents the most mature candidate in global clinical development and launched its Phase II clinical trial in Bangladesh in December 2025.

The ChAdOx1 vector system has been well validated during the development and licensure of the Oxford-AstraZeneca COVID-19 vaccine, with its safety, tolerability, and immunogenicity comprehensively confirmed in large-scale human trials. Preclinical challenge experiments in non-human primates have verified that a two-dose immunization schedule of ChAdOx1 NipahB is capable of inducing potent, stable protective immune responses against Nipah virus infection [86]. This candidate has obtained the Priority Medicines (PRIME) qualification from the European Medicines Agency (EMA), enabling accelerated regulatory review and approval procedures. Supported by the Coalition for Epidemic Preparedness Innovations (CEPI), the Serum Institute of India has manufactured 100,000 vaccine doses for emergency reserve, which can be rapidly deployed for on-site vaccination and epidemic intervention once Nipah virus outbreaks occur [87].

PHV02 is a recombinant VSV-based Nipah virus vaccine constructed using the identical technical platform of Ervebo, a clinically licensed Ebola vaccine with proven safety and therapeutic efficacy [88]. A prominent strength of PHV02 lies in its capacity to trigger rapid protective immunity after a single immunization, rendering it highly adaptable to emergency interventions for sporadic Nipah virus outbreaks. This candidate has successfully completed Phase I clinical assessment with a favorable safety profile and no serious adverse events, and its subsequent Phase II trial is scheduled to be conducted in Bangladesh. Owing to its single-dose rapid protection feature, PHV02 is highly compatible with ring vaccination, the pivotal intervention strategy for containing local Nipah virus transmission, and can effectively improve the response efficiency and overall performance of emergency epidemic management [89].

mRNA-1215 is an innovative chimeric mRNA vaccine encoding pre-fusion F/G chimeric antigens of Nipah virus, which targets two critical surface glycoproteins responsible for viral attachment and host cell entry. Preclinical experimental data have demonstrated that this vaccine elicits broader-spectrum and stronger humoral immune reactions, as well as robust and long-lasting antigen-specific T-cell responses, compared with conventional vaccines that only target the single G glycoprotein. Such superior immune responses confer enhanced and comprehensive protective potential against viral infection [90]. Currently undergoing Phase I clinical evaluation, mRNA-1215 inherits the core advantages of mRNA technology, including streamlined research workflows, rapid antigen modification and iteration, and scalable large-scale industrial production [91].

The Hendra virus soluble G protein vaccine (HeV-sG-V) is a recombinant subunit vaccine that delivers stable and reliable protective effects against Henipavirus infection across multiple animal challenge models [92]. Finished Phase I clinical studies have validated that the vaccine satisfies the preset evaluation criteria for human safety, tolerability, and immunogenicity. By targeting the highly conserved G protein of Hendra virus, HeV-sG-V can induce cross-protective immunity against two major zoonotic pathogens in the Henipavirus genus: Nipah virus and Hendra virus. This candidate holds great promise as a universal pan-Henipavirus vaccine, providing novel technical support for routine prevention and emergency control of henipavirus-associated zoonotic diseases [93].

The four clinically progressed Nipah virus vaccine candidates exhibit distinct differences in structural design, research maturity, immunization regimens, immune response kinetics, and practical application scenarios, with respective advantages and inherent limitations. ChAdOx1 NipahB benefits from a mature adenoviral vector platform and standardized regulatory approval pathways; nevertheless, pre-existing vector-specific immunity in general populations and the mandatory two-dose immunization procedure hinder its rapid deployment and large-scale application during sudden epidemic outbreaks. As a fast-acting single-dose vaccine adaptable to ring vaccination strategies, PHV02 is well suited for emergency epidemic disposal, whereas the potential neurotoxicity derived from the replication-competent viral vector requires further verification and systematic risk evaluation via large-scale clinical trials. mRNA-1215 possesses high production efficiency and promising industrialization prospects, while no Phase I clinical data have been publicly released so far. Additionally, its strict reliance on cold-chain logistics and storage conditions limits its popularization in resource-scarce epidemic regions, necessitating further technical optimization and process upgrading. HeV-sG-V exhibits the most favorable safety profile with solid Phase I clinical validation results, yet its two-dose immunization scheme and relatively high manufacturing costs restrict its applicability in emergency mass immunization and large-scale population prevention.

Vaccines developed from different technical platforms possess unique and non-substitutable application values in epidemic prevention. ChAdOx1 NipahB is mainly applicable to routine preventive immunization for high-risk populations. With the advantages of rapid immune activation and scalable mass production, PHV02 and mRNA-1215 are highly suitable for emergency response to unexpected outbreaks. Featuring superior safety performance, HeV-sG-V can provide high-grade immune protection for immunocompromised patients and other vulnerable groups at high infection risk.

From the perspective of industrial transformation and global popularization, all four clinical-stage vaccine candidates face a universal research bottleneck: the lack of large-sample real-world evidence supporting their protective efficacy in human populations. Naturally occurring Nipah virus epidemics generally present sporadic distribution and small-scale outbreaks, which makes traditional large-sample, multicenter, randomized controlled Phase III clinical trials technically difficult and practically infeasible for efficacy assessment.

To overcome this technical barrier, a feasible and widely recognized research strategy has been established in global academic and regulatory fields. Specifically, immune protection thresholds derived from standardized non-human primate models can be adopted as surrogate endpoints for vaccine registration and licensing, which is well exemplified by the Animal Rule implemented by the U.S. Food and Drug Administration (FDA). Furthermore, utilizing global pre-established vaccine emergency reserves to conduct targeted ring vaccination during local outbreaks enables continuous accumulation of real-world efficacy data. This innovative strategy facilitates the construction of a complete translational loop covering iterative regulatory optimization, preclinical animal verification, and real-world clinical validation.

In summary, compared with the single-platform vaccine development model, the diversified research strategy based on multiple complementary technical platforms and vaccine reserves represents a more scientific and practical solution for global Nipah virus epidemic prevention and control. This multi-dimensional system can fully meet the demands of routine prevention, sudden epidemic disposal, and targeted protection for special high-risk groups, thereby greatly optimizing the comprehensive defense system against henipavirus threats.

In addition to the above vaccines progressing into clinical stages, numerous Nipah virus candidate vaccines, measles virus vector vaccine (fMV-NiV-F/G), virus-like particle vaccine (NiV M-F-G VLP), and multi-epitope vaccine targeting L protein, are currently under diverse preclinical research phases [94,95,96,97,98,99]. Table 2 systematically sorts and compares the research progress of both clinical and preclinical Nipah virus vaccines, with a detailed focus on antigen design strategies, characteristics of humoral and cellular immune responses, protective outcomes of animal challenge assays, and overall developmental status.

## 5. Regulatory Adaptation and Implementation Strategy Recommendations for Nipah Virus Vaccine Development

NiV exhibits sporadic transmission patterns, a high case fatality rate, and limited human-to-human spread. Conventional vaccine research and development (R&D) paradigms and regulatory frameworks fail to fully accommodate the translational and emergency application demands of NiV vaccines. Adaptive revisions to the full-spectrum R&D and regulatory review system are therefore imperative. Grounded in virology, vaccinology, and regulatory science principles, this work proposes comprehensive implementation strategies to consolidate global industrial, academic, and regulatory resources, streamline vaccine translational efficiency, and improve the public health accessibility of safe and efficacious NiV vaccines.

### 5.1. Establishment and Standardization of Correlates of Vaccine Protection

#### 5.1.1. Current Status

Studies have demonstrated that vaccination with the PHV02 vaccine confers favorable prognostic outcomes, including animal survival and suppression of clinical infection. Seroconversion of neutralizing antibodies (neutralizing antibody titers ≥ 1:5 induced by PHV02 are defined as a surrogate marker for animal survival) can serve as a potential protective biomarker [87]. The HeV-sG-V vaccine has established correlates of protection (CoP) threshold values against Nipah virus based on humoral immune readouts. Total ELISA IgG antibody titers and FRNT50 neutralizing antibody titers act as stable and reliable predictors of survival outcomes, with neutralizing antibody-associated metrics representing the optimal mechanistic protective indicators [100].

Nevertheless, vaccination platforms differ fundamentally in the pathways and magnitude of immune activation they elicit. Correlates of protection exhibit strict vaccine-, pathogen- and species-specificity. Even vaccines targeting the identical pathogen via distinct platforms may rely on entirely divergent immune mechanisms and threshold levels to mediate protective efficacy. Accordingly, further in-depth investigations are required to characterize the generalizability and standardization of CoPs across disparate vaccine platforms. Standardized platforms for NiV-neutralizing antibody detection and globally validated quantitative thresholds for correlates of protection (CoP) remain absent. These technical gaps preclude cross-platform comparisons of immunogenicity data across diverse NiV vaccine candidates, constrain the adoption of immunobridging-based regulatory frameworks, and hinder the standardized implementation of accelerated approval pathways for NiV vaccines.

#### 5.1.2. Optimization Recommendations

(1) Establishing a standardized comprehensive evaluation system for vaccine immunogenicity

International regulatory authorities should initiate multi-center collaborative studies across global academic and industrial institutions. Using well-validated non-human primate models as the core evaluation system, these studies systematically characterize humoral and cellular immune responses elicited by distinct vaccine platforms. Identification of immunological biomarkers tightly associated with antiviral protection and definition of threshold values (e.g., the minimum protective neutralizing antibody titer) enables the establishment of a standardized, reproducible immunogenicity evaluation framework for NiV vaccines.

(2) Promoting standardization and homogenization of neutralizing antibody detection systems

Global calibration and open sharing of NiV reference materials, including standardized reference sera and pseudovirus strains, are essential. Cross-laboratory standard operating procedures (SOPs) for neutralizing antibody detection minimize data heterogeneity derived from inconsistent assay systems and experimental conditions. These measures ensure the accuracy, reproducibility, and comparability of NiV vaccine immunogenicity data, providing robust standardized evidence for immunobridging-based regulatory evaluation.

### 5.2. Global Resource Coordination and Collaborative Optimization of Alternative Regulatory Pathways

#### 5.2.1. Current Status

The sporadic nature of NiV transmission and the scarcity of natural clinical cases preclude conventional large-scale, multi-center phase III confirmatory clinical trials, rendering traditional efficacy-based marketing approval unfeasible. Although alternative regulatory pathways such as the Animal Rule are available globally, major regulatory bodies, including the U.S. Food and Drug Administration (FDA), European Medicines Agency (EMA), and Australian Therapeutic Goods Administration (TGA), maintain divergent implementation criteria and technical specifications, leading to substantial regulatory inconsistencies and translational barriers.

#### 5.2.2. Optimization Recommendations

(1) Facilitating global regulatory consensus-building and mutual recognition of review results

Under the regulatory coordination framework of the World Health Organization (WHO), global regulatory authorities shall reach a unified scientific consensus on key regulatory endpoints for NiV vaccines, encompassing animal challenge model criteria, immunobridging trial design, primary efficacy outcomes, and safety evaluation metrics. Harmonization of these standards eliminates redundant animal and clinical testing and facilitates a global collaborative mechanism for synchronous review and cross-jurisdictional result recognition, thereby accelerating worldwide simultaneous registration and marketing of NiV vaccines.

(2) Optimizing the allocation of high-level BSL-4 biosafety resources

Live-virus NiV challenge assays exclusively rely on biosafety level 4 (BSL-4) laboratories, which are globally scarce and unevenly distributed. The development of a regional BSL-4 collaborative network enables centralized integration of high-level biosafety resources, supporting standardized core challenge protection and efficacy verification assays for NiV vaccines. This centralized model avoids redundant low-level experimentation and resource wastage, and improves the reliability, efficiency, and authority of key pharmacodynamic and safety datasets.

### 5.3. Construction of Emergency Vaccine Reserve System and Dynamic Collection of Real-World Data

#### 5.3.1. Current Status

NiV outbreaks occur sporadically within restricted geographic regions, resulting in limited commercial prospects for NiV vaccine development. Pharmaceutical manufacturers therefore lack economic incentives to conduct large-scale phase III confirmatory trials. Furthermore, routine mass vaccination in non-endemic areas presents poor cost-effectiveness, and conventional universal vaccination strategies are incompatible with NiV-specific epidemic prevention and control demands.

#### 5.3.2. Optimization Recommendations

(1) Establishing a multilateral collaborative emergency reserve mechanism for NiV vaccines

Supported by global public health initiatives such as the Coalition for Epidemic Preparedness Innovations (CEPI), a dedicated strategic reserve bank for NiV emergency vaccines can be established. The integration of advance market commitment (AMC) mechanisms stabilizes industrial R&D incentives. NiV vaccine candidates that complete phase II clinical evaluation, demonstrate favorable benefit-risk profiles, and obtain conditional marketing authorization qualify for scaled production and strategic stockpiling, ensuring rapid deployment and immediate emergency intervention during sporadic NiV outbreaks.

(2) Implementing ring vaccination and embedding real-world research systems

During localized NiV outbreaks, targeted ring vaccination of confirmed case contacts and high-risk populations effectively interrupts human-to-human transmission chains. Prospective, standardized real-world study (RWS) protocols enable systematic collection of field-based data regarding vaccine efficacy, adverse event profiles, and population-specific safety outcomes. The resulting real-world evidence supports subsequent full approval, indication expansion, and optimization of immunization regimens for NiV vaccines.

### 5.4. Mitigation of Biosafety-Derived Restrictions on Vaccine R&D Efficiency

#### 5.4.1. Current Status

As a highly pathogenic agent, NiV requires all live-virus manipulation and etiological validation experiments to be conducted in BSL-4 facilities. This stringent biosafety barrier limits institutional accessibility to NiV research, impedes iterative progress in antigen design, candidate strain modification, and formulation optimization, and delays the overall translational timeline of NiV vaccine development.

#### 5.4.2. Optimization Recommendations

(1) Establishment of a Non-live Virus Alternative Technical System to Reduce Dependence on BSL-4 Platforms

A low-risk, high-throughput non-live virus in vitro alternative system effectively reduces NiV vaccine R&D dependence on scarce BSL-4 platforms. Well-validated approaches, including pseudovirus neutralization assays, cell-based screening platforms, and organoid infection models, replace conventional live virus operations to support preliminary antigen screening, candidate molecular evaluation, and formulation efficacy prediction. Massive candidate optimization is completed via low-biosafety alternative systems, while only essential etiological verification is performed in BSL-4 laboratories. This strategy reduces BSL-4 experimental workload and alleviates NiV vaccine R&D stagnation caused by high-level platform resource shortages.

(2) Construction of a Hierarchical and Progressive R&D Mode to Accelerate Early-Stage Iteration

A hierarchical closed-loop R&D framework accelerates NiV vaccine early-stage iteration. This tiered system decouples routine preliminary exploration from core verification, conducting high-throughput antigen design, strain modification, and formulation optimization in low-to-medium biosafety laboratories to rapidly eliminate invalid candidates. Only high-potential vaccine candidates proceed to BSL-4 live virus verification. High-level experimental data guides further scheme optimization, forming a virtuous loop of “preliminary iteration–precise verification–upgrade optimization” and substantially promoting efficient early-stage vaccine R&D progress.

## 6. Conclusions

As a highly pathogenic zoonotic pathogen with an extremely high case fatality rate, NiV has continuously caused recurrent small, sporadic epidemic events throughout South and Southeast Asian regions. The 2026 West Bengal outbreak further highlights its persistent public health threats and the critical lack of fully licensed NiV vaccines and specific antiviral therapeutics. Accordingly, to address this unmet clinical and public health demand, future NiV vaccine development may benefit from prioritizing rational antigen design, multi-platform technical innovation, and translational regulatory optimization. Specifically, advanced immunogen strategies, including pre-F protein stabilization, chimeric G/F antigen construction, and conserved L-protein multi-epitope design, could be further explored to counteract NiV-mediated immune evasion. Such structural and immunological refinements would support the development of broader-spectrum, high-efficacy vaccine candidates. Furthermore, the integration and iterative optimization of four well-established clinical-stage platforms, including viral vector, mRNA, recombinant subunit, and live-attenuated vaccines, may facilitate the establishment of a more systematic and complementary research framework to resolve key bottlenecks in conventional NiV vaccine development.

Building on the above technical advances, this review systematically compares the technical profiles, applicable scenarios, and inherent limitations of current clinical-grade NiV vaccine candidates, indicating that differentiated R&D and deployment strategies are preferable for distinct technical routes. Specifically, the adenovirus-vectored ChAdOx1 NipahB features robust technical maturity and stable immunogenicity, making it suitable for routine prophylaxis among high-risk populations; nevertheless, further optimization is warranted to mitigate the impacts of pre-existing vector immunity and the constraints of its two-dose regimen. In contrast, the single-dose VSV-based PHV02 induces rapid protective immunity and is well suited for emergency ring vaccination, while large-scale clinical validation is still required to clarify its potential vector-associated safety risks. Meanwhile, the chimeric mRNA-1215 vaccine, with flexible antigen iteration and scalable manufacturing capacity, could be prioritized for emergency stockpiling, and formulation optimization may overcome cold-chain dependence to expand its applicability in resource-limited endemic regions. Additionally, the HeV-sG-V subunit vaccine exhibits superior safety and broad cross-protection against henipaviruses for vulnerable populations, whereas cost reduction and accelerated immune induction would facilitate its large-scale field deployment. Finally, preclinical VLP and L-protein multi-epitope candidates merit continuous advancement to enrich the NiV vaccine pipeline and enhance resistance against strain-specific immune escape.

Despite substantial progress in NiV antigen design and vaccine platform optimization, NiV’s sporadic epidemic patterns, low endemic incidence, and scarcity of large-scale outbreak data render conventional Phase III-dependent licensing pathways impractical. As such, adaptive regulatory frameworks tailored to NiV’s unique epidemiological characteristics are highly necessary. In particular, global regulatory authorities may consider standardizing protective correlate thresholds, strengthening cross-border regulatory collaboration, improving emergency vaccine reserve systems, and optimizing the allocation of limited BSL-4 biosafety resources. Collectively, such adaptive regulatory refinements can effectively relieve translational barriers, standardize emergency approval procedures, and improve the overall efficiency of NiV outbreak intervention.

To further overcome the aforementioned technical and regulatory limitations, future NiV vaccine research and translational industrialization would benefit from cross-platform integration and technical iteration, so as to compensate for the inherent defects of single-platform designs and develop safer, more field-adaptable vaccine candidates. Three promising research directions are highlighted in this review. Firstly, multi-platform complementary integration deserves further exploration; combining the rapid immune activation of chimeric mRNA antigens, durable immune memory elicited by viral vector platforms, and high safety profile of subunit vaccines may generate composite candidates that balance emergency intervention and long-term routine prevention. Secondly, precision antigen design targeting conserved L-protein functional domains and stabilized pre-F/G epitopes may advance the development of pan-lineage vaccines capable of tolerating NiV-BD sublineage drift and viral immune evasion. Thirdly, continuous industrial optimization, including the development of cold-chain-independent mRNA formulations, low-cost scalable subunit production protocols, and systematic safety evaluation of live-attenuated vectors, could substantially improve vaccine adaptability in resource-scarce endemic regions.

Beyond technical innovation, standardized regulatory construction is equally essential, given that a globally unified full-cycle evaluation system for NiV vaccines remains incomplete and requires further harmonization. Specifically, international organizations and national regulatory agencies may consider establishing consensus-based protective correlate standards and unified Animal Rule evaluation criteria, optimizing cross-border synchronous review mechanisms, and streamlining redundant evaluation procedures to accelerate global vaccine registration. Furthermore, continuous collection and retrospective analysis of real-world evidence from ring vaccination campaigns will help validate and refine vaccine efficacy and safety, establishing a closed-loop translational pipeline covering rational antigen design, preclinical verification, adaptive approval, and field validation. In summary, future NiV vaccine development can leverage diversified technical layouts, refined immunogen design, and adaptive regulatory strategies to mitigate core developmental bottlenecks. Ultimately, advances in these directions will strengthen global preparedness against NiV and other high-risk zoonotic pathogens and further improve the resilience of global public health defense systems.

## Figures and Tables

**Table 1:** Quantitative summary of NiV transmission pathways and at-risk populations.

		Quantified Case Proportions		Region	Lineages	Data Source
**Major transmission pathways**	Foodborne spillover	Responsible for 45.72% of cases (95% CI: 40.33–51.19)		Bangladesh, 2001–2023, *n* = 339	NiV-BD Seasonal (December–April)	[6,7,79,80]
	Human-to-human transmission	66.37% of cases associated with cluster events (225/339; 95% CI: 61.08–71.40)	51% of secondary cases occurring in household/caregiver settings		NiV-BD Seasonal (December–April)	
			9% in healthcare workers			
			The basic reproduction number R_0_ in community settings is estimated at <1.0 (R_0_ ≈ 0.3–0.5)			
	Animal intermediate spillover	The symptomatic infection rate was 51% (25/43) among high-intensity pig-exposed individuals		Malaysia context, 1998–1999, *n* = 265 (40% CFR), in a pig farm cohort	NiV-MY	[81,82,83]
		The symptomatic infection rate was 21% (5/24) among minimal-exposure individuals				
	Direct bat-to-human (non-foodborne)	The bat seroprevalence was 15.2% (95% CI: 10.74–20.56) in outbreak-adjacent roosts		Bangladesh	NiV-BD	[67,84]
**Population at risk**	Healthcare workers	Pooled seroprevalence 0.08% (95% CI: 0.00–0.72%), with a pooled case fatality ratio of 73.52% (95% CI: 34.01–99.74)		Malaysia, Singapore, Bangladesh, India and the Philippines	NiV-BD Seasonal (December–April)	[75]
	Pig industry workers	Symptomatic infection rate 51% among high-intensity pig-exposed		Malaysia	NiV-MY	[81]
		Symptomatic infection rate 21% among minimal-exposure individuals				
	Date palm sap harvesters/consumers	73.1% of outbreak community participants had sap consumption habits; adjusted odds ratio for NiV infection among raw sap consumers = 3.96 (95% CI: 3.5–4.4)		Bangladesh	NiV-BD Seasonal (December–April)	[67,79,85]
	Rural/peri-urban residents	General population seroprevalence 0–12.5%; presence of bat roosts within 5 km of village confers an aOR = 1.18 (95% CI: 1.02–1.37) for NiV spillover		South Asia	NiV-BD	[68]

**Table 2:** Summary and comparison of mainstream Nipah virus vaccine candidates in preclinical and clinical trials.

Developer	Vaccine Name	Antigen Design	Challenge Protection	Animal Model/Immunization Schedule	Humoral Immunity	Cellular Immunity	Clinical Progress/Outcomes	References
University of Oxford	ChAdOx1 NiVb	A recombinant adenovirus vaccine constructed using replication-deficient chimpanzee adenovirus ChAdOx1 as the vector, inserted with codon-optimized G protein gene of NiV Bangladesh strain	Complete protection observed in all immunized macaques	Adult African green monkeys; intramuscular administration, 2 doses	NiV-G-specific IgG was detectable as early as 14 days post-primary immunization; booster immunization significantly increased antibody titers. Neutralizing antibodies were detected on Day 14, with a marked elevation after booster vaccination.	A robust NiV-G-specific T cell response was induced after single-dose immunization; booster immunization further significantly enhanced T cell responses (*p* = 0.0271), which were substantially higher than those in the empty vector control group	Enrollment of all 61 subjects was completed in 2025, and the trial is currently in the long-term follow-up phase. A Phase II clinical trial is planned to be launched in Bangladesh.	[86,87]
Public Health Vaccines LLC (PHV), USA	PHV02	A replication-competent recombinant live viral vaccine based on attenuated vesicular stomatitis virus (rVSV) with its virulent G gene deleted, co-expressing Ebola virus GP protein and G protein of NiV Bangladesh strain	All animals in the high- and medium-dose groups survived after virus challenge	African green monkeys; single intramuscular injection	1. Geometric mean titer (GMT) of neutralizing antibodies was 71.3. 2. For animals immunized 14 days before challenge: GMT = 10, and 67% of animals had antibody titers ≥ 1:5. 3. For animals immunized 7 days before challenge: GMT = 4, and antibodies were only detectable in 33% of animals.	No available data	Phase I clinical results showed that single intramuscular injection rapidly induced NiV-specific neutralizing antibodies, with superior efficacy in the high-dose group. A Phase II trial will be conducted in epidemic areas of Bangladesh.	[88,89]
Moderna & Vaccine Research Center, National Institutes of Health (NIH-VRC), USA	mRNA-1215	Pre-F protein and G protein of NiV	/	Female CB6F1/J mice; 2 intramuscular doses	1. Binding antibodies: Titers of pre-F and pre-F/G groups all exceeded 1:65,000 with no obvious dose-dependent difference; post-F antibody titers increased in a dose-dependent manner. 2. Neutralizing antibodies: Hex G and pre-F/G exhibited comparable neutralizing potency, which was 10-fold higher than that of pre-F alone. G protein served as the major neutralizing target. At the same dosage, the neutralizing activity of mRNA vaccine was 40–60% higher than that of protein vaccine.	1. pre-F/G chimeric antigen elicited stronger follicular helper T (Tfh) cell and effector T cell responses than Hex G, with a broader immune spectrum. 2. The mRNA vaccine effectively activated CD4^+^ and CD8^+^ T cells, dominated by F protein-specific T cells. 3. At the same dosage, the mRNA vaccine induced stronger CD8^+^ T cell responses than the protein vaccine, facilitating the elimination of infected cells.	Phase I clinical trial enrollment has been completed.	[90,91]
University of Texas Medical Branch, Profectus BioSciences & Uniformed Services University of the Health Sciences, USA	HeV-sG-V	A human subunit vaccine formulated with soluble glycoprotein of Hendra virus (HeV-sG) as the immunogen and adjuvanted with Alhydrogel (aluminum hydroxide)	1. All animals in the conventional immunization group survived. 2. Most animals obtained full protection when challenged at 7 days post-immunization.	African green monkeys; single or two intramuscular doses	1. Binding antibodies: On the day of challenge, antibody titers in the control group were below 100 EU, while all immunized groups developed high-titer HeV-sG binding antibodies. Titers in the single-dose group were slightly lower than those in the booster group, and antibody levels of all immunized animals further increased after challenge. 2. Neutralizing antibodies: On the day of challenge, anti-HeV titers ranged from 320 to 2560, and anti-NiV-B titers ranged from 10 to 160.	No available data	Phase I clinical trial enrollment has been completed.	[92,93]
CSIRO Australia, PATH & Auro Vaccines	HeV-sG	Soluble G glycoprotein of Hendra virus	Full protection in ferrets and green monkeys (protection duration ≥ 14 months); insufficient protection in pigs	Ferrets, green monkeys, cats and pigs; intramuscular injection with prime-boost regimen	The maximum antibody titer in cats reached 20,000 and persisted for 2 months; long-lasting antibodies were detected in green monkeys and ferrets.	Favorable cellular immune responses were observed in primates and ferrets; weak cellular immune memory was detected in pigs.	preclinical	[94,95]
National Institutes of Health (NIH), USA	NiV pre-F/G	Covalently combined pre-F protein and G protein	Complete protection against lethal virus challenge	Mice and golden hamsters; intramuscular injection with prime-boost regimen	Higher antibody titers compared with vaccines using F protein or G protein alone	Activated Tfh cells and effector T cells, inducing balanced immune responses.	preclinical	[96]
U.S. Army Medical Research Institute of Infectious Diseases (USAMRIID)	rVSV-ΔG-NiVB-G/F	VSV vector with endogenous G gene deleted, expressing G and F proteins of NiV Bangladesh strain	Full protection in all animal models	Ferrets, hamsters and African green monkeys; single intramuscular injection	Antibody titer of 80 in ferrets at 7–8 days post immunization; titer of 100 in hamsters at 32 days post immunization. Titers reached 1:640 in the G protein group and 1:160~640 in the F protein group of green monkeys.	Activated both innate and adaptive immunity, with robust CD8^+^ T cell activation.	preclinical	[97]
The University of Tokyo, Japan	rMV-NiV-F/G	Attenuated measles virus vector expressing NiV F and G proteins	100% survival rate; no virus detected in visceral organs	Hamsters and green monkeys; single dose via oral, intranasal or intramuscular administration	The peak antibody titer reached 1600 within 14 days.	Insufficient data on cellular immunity.	preclinical	[98]
U.S. Army Medical Research Institute of Infectious Diseases (USAMRIID)	NiV M-F-G VLP	Nucleic acid-free virus-like particles (VLPs) self-assembled by NiV M, F and G proteins	Complete protection against lethal virus challenge	BALB/c mice and golden hamsters; single or three doses	Antibody titers in mice exceeded 80 at 35 days post-immunization; IgM was produced first, followed by IgG conversion in hamsters.	Activated innate immunity, dominated by B cell responses.	preclinical	[99]

## Data Availability

Data are contained within the article.

## Abbreviations

The following abbreviations are used in this manuscript:

BSL-4	Biosafety Level 4
CEPI	Coalition for Epidemic Preparedness Innovations
EMA	European Medicines Agency
FDA	Food and Drug Administration
F	Fusion glycoprotein
G	Attachment Glycoprotein
GMT	Geometric Mean Titers
HeV	Hendra virus
Ifnar^−^/^−^	Type I Interferon Receptor-deficient
ISGs	Interferon Stimulated Genes
L	Long Polymerase
LNP	Lipid Nanoparticle
M	Matrix Protein
MV	Measles virus
MDA5	Melanoma Differentiation Associated Protein 5
NHPs	Non-Human Primates
NIAID	National Institute of Allergy and Infectious Diseases
NiV	Nipah virus
NiV MY	Nipah virus Malaysia lineage
NiV-BD	Nipah virus Bangladesh lineage
N	Nucleocapsid
P	Phosphorprotein
PBMCs	Peripheral Blood Mononuclear Cells
PHV	Public Health Vaccines
pre-F	Prefusion Conformation of F protein
PRIME	Priority Medicines
PRNT	plaque reduction neutralization test
rVSV	Replication-deficient Recombinant VSV
R&D	research and development
sG	Soluble G Protein
Tfh	Follicular Helper T
VCTD	C Terminal Domain of the V protein
VLP	Virus-like Particle
VN	virus neutralization
VSV	Vesicular Stomatitis Virus
WHO	World Health Organization
